# Performance of the One Health platform in zoonotic disease surveillance in Guinea

**DOI:** 10.3389/fpubh.2025.1634641

**Published:** 2025-09-19

**Authors:** Emile F. Bongono, Sidikiba Sidibé, Castro G. Hounmenou, Aminata Mbaye, Kadio J. J. O. Kadio, Aly B. Nabé, Maladho Diaby, Foromo Timothée Beavogui, Mohamed Idriss Doumbouya, Alexandre Delamou, Abdoulaye Touré, Alioune Camara, Alpha Kabinet Keita

**Affiliations:** ^1^Centre de Recherche et de Formation en Infectiologie de Guinée (CERFIG), Université Gamal Abdel Nasser de Conakry, Conakry, Guinea; ^2^Département de Maladies Infectieuses et Parasitaires de l' Institut Supérieur des sciences et de Médecine Vétérinaire (ISSMV) de Dalaba, Dalaba, Guinea; ^3^Chair of Public Health, Department of Medical Sciences, Faculty of Health Sciences and Techniques, Gamal Abdel Nasser University of Conakry, Conakry, Guinea; ^4^Département de l'Informatique, Université de Labé, Labe, Guinea; ^5^Direction Nationale des Services Vétérinaire, Ministère de l'Agriculture et de l'Élevage, Conakry, Guinea

**Keywords:** One Health approach, platform performance, zoonotic disease, epidemiologic surveillance, Guinea

## Abstract

**Introduction:**

Zoonoses are a major global health threat, especially in low-income countries, due to their prevalence and emergence. Repeated outbreaks emphasize the need for integrated, multisectoral surveillance. While the One Health approach is essential, its implementation faces major barriers. Tools like JEE and OH-EpiCap help assess and improve these systems. This study aims to assess the functioning and effectiveness of regional One Health platforms in Guinea.

**Methods:**

A cross-sectional study was conducted across the eight administrative regions of Guinea to evaluate the performance of regional One Health (OH) platforms. Data were collected through structured interviews with 160 stakeholders involved in zoonotic disease surveillance, preparedness, and response. The evaluation focused on several key components: coordination; case recording and disease detection; epidemic preparedness and response; mobilization of material resources; stakeholder training; and financing mechanisms. Regional performance was assessed using the standardized evaluation tool developed by the Africa CDC. A comparative analysis was performed using radar charts to identify performance gaps between regions and to highlight disparities in the implementation of the One Health approach.

**Results:**

The overall One Health performance score in Guinea was 41%, indicating a limited level of implementation at the national scale. None of the eight assessed regions reached the 60% performance threshold. Indicator-level analysis revealed significant heterogeneity across regions. Conakry demonstrated strong performance in the domain of legislation (89%), whereas all regions exhibited weak capacities in the mobilization of material resources (9%), highlighting a major cross-cutting challenge. Regional performance scores varied considerably, with particularly low levels observed in Labé, Kindia, and Faranah (33%), underscoring major disparities in the implementation of the One Health framework.

**Conclusion:**

This study identified critical gaps in the performance of Guinea's One Health platforms, notably in resource mobilization and regional disparities. Strengthening local capacities, harmonizing practices, and improving multi-sectoral coordination are essential. Using the Africa CDC assessment tool revealed actionable insights to inform policy and investment. These findings emphasize the urgent need to reinforce One Health implementation amid persistent zoonotic threats in the country.

## Introduction

Zoonoses are communicable diseases transmitted between animals and humans, accounting for ~60% of human infectious diseases and 75% of emerging infections (1–3). They therefore represent a major threat to global public health (4–7). Fragile interactions between humans, animals, ecosystems, and health systems exacerbate the challenges faced by low-income countries. Globally, outbreaks of infectious diseases such as Ebola, Marburg, H5N1, SARS-CoV, and Lassa fever underscore the need for robust surveillance and cross-sectoral collaboration (8–11). These outbreaks expose critical weaknesses in surveillance systems and highlight the importance of an integrated approach for timely detection and effective response (12–14).

Epidemics such as HIV/AIDS and COVID-19 have disrupted global health and economic systems (8, 9). Effective surveillance is critical for controlling zoonoses (6, 13) and recurrent outbreaks—including Ebola, COVID-19, avian influenza, and rabies—underscore the urgent need to strengthen surveillance and response systems (8, 9, 15, 16). The One Health (OH) approach, which integrates human, animal, and environmental health, is essential for addressing these complex challenges (17–19). This approach enhances zoonotic surveillance and facilitates cross-sectoral collaboration (11, 17, 20), It has been adopted in many countries to improve coordination and preparedness efforts (12, 21–23).

However, the implementation of the One Health (OH) approach faces several obstacles, including limited cross-sectoral collaboration, inadequate infrastructure, and fragmented data systems (12, 21–23). Understanding these barriers is essential for developing effective strategies to strengthen OH initiatives (24, 25). Additional challenges include the lack of investigative tools, insufficient funding, the absence of a formal institutional framework, and inadequate training of personnel involved in OH platforms (3, 26).

The assessment of One Health (OH) platforms relies on key indicators such as early detection of zoonotic outbreaks, effective coordination of response activities, and maintaining sustainability despite limited resources (5, 20). Several tools, including the Joint External Evaluation (JEE) (6, 26–28), One Health OH-EpiCap, and the Performance of Veterinary Services (PVS) pathway, are commonly used to evaluate OH platform monitoring. These tools focus on aspects such as resource availability, data collection and sharing, and data analysis and interpretation (17, 29–31). However, economic constraints often limit their widespread application.

Guinea's rich biodiversity makes it a hotspot for emerging zoonoses (32). The Ebola outbreak exposed significant weaknesses in Guinea's surveillance and response capacities (33, 34), underscoring the urgent need to establish and implement One Health (OH) platforms in the country. These platforms, led by local stakeholders, play a crucial role in coordinating responses to health crises and promoting preventive measures. Despite strong commitment from the Guinean government, enhanced cooperation and collaboration across sectors remain essential to fully realize the potential of the One Health (OH) approach (35).

In response to these needs, the National One Health Platform (PNOH) was established in Guinea under the supervision of the Ministry of Health. Its main objective is to prevent, detect, and respond to emerging and re-emerging diseases with pandemic potential by adopting a multisectoral approach that integrates human, animal, and environmental health sectors.

The One Health initiative in Guinea seeks to strengthen collaboration among various governmental actors, as well as technical and financial partners, to optimize health surveillance and resource mobilization for public health emergencies. This initiative is grounded in internationally recognized frameworks, including the International Health Regulations (IHR), the Performance of Veterinary Services (PVS), and the Global Health Security Agenda (GHSA).

To fulfill its mandate, the OH platform in Guinea is organized through several key bodies, including a steering committee, a multisectoral technical coordination committee, a permanent secretariat, Technical Working Groups (TWGs), and Emergency Operations Centers (EOCs).

Nevertheless, despite these organizational structures and efforts, OH platforms continue to face challenges in meeting intervention standards due to limited funding, insufficient integration of assessment tools, and fragmented institutional frameworks. This study therefore aims to assess the capacities of actors involved in zoonosis surveillance within these OH platforms.

## Materials and methods

### Scope of study

This study was conducted in the Republic of Guinea, located in West Africa between latitudes 7° and 12° North and longitudes 8° and 15° West. Covering an area of 245,852 km^2^ and with an estimated population of 14 million, Guinea experiences a tropical climate characterized by distinct rainy and dry seasons influenced by the Harmattan wind (36). The country is rich in biodiversity, hosting diverse flora and fauna. Intensive agricultural activities by local populations increase interactions between humans, domestic animals, wildlife, and the environment, thereby creating conditions conducive to the emergence and spread of zoonotic diseases (37). The study covered all eight administrative regions of Guinea—Conakry, Boké, Kindia, Mamou, Labé, Kankan, Faranah, and N'zérékoré—selected due to their history of zoonotic epidemics such as Ebola, rabies, and other animal-transmitted infections (37). In response to these risks, Guinea has adopted the One Health (OH) approach to effectively manage zoonotic threats (38). Consequently, a One Health platform has been established in each region at the prefecture level within their respective jurisdictions.

### Study design and period

This was a cross-sectional study conducted between May and June 2023, involving 160 identified stakeholders.

### Population and sampling

A purposive sampling approach was employed, targeting actors actively involved in the activities of the regional One Health (OH) platforms. Regional OH focal points were first identified, and together with other relevant stakeholders, they were invited to participate in regional workshops where individual questionnaires were administered.

It is important to note that the number of participants corresponds to the actual size of the regional OH platforms in Guinea. The aim was not to achieve statistical representativeness of the entire national health workforce, but rather to ensure functional representativeness of key actors engaged in intersectoral coordination and integrated zoonotic disease surveillance.

### Participants

Participants were primarily drawn from the three core sectors of human, animal, and environmental health. These included veterinarians, human and animal health technicians, disease control officers, physicians, epidemiologists, laboratory technicians, biologists, data managers, forestry and natural resource conservation officers, environmental agents, and local elected officials.

### Data collection

Data were collected using standardized questionnaires inspired by tools developed by Africa CDC and the World Health Organization (WHO) (39, 40). The questionnaire was adapted to the Guinean context through a document review and validation process by local experts. It was structured around seven key indicators: Legislation (LID): Existence of regulatory texts or manuals defining the mechanisms for integrated disease surveillance.

Epidemic Detection and Documentation (EDEIPD): Presence of documentation and early warning mechanisms for epidemic outbreaks.

Preparedness (PREID): Existence of mechanisms for epidemic preparedness and response.

Training of Actors (FPID): Existence of disease surveillance training programs or trained personnel involved in the implementation of OH platform activities.

Material Resources (RMID): Availability of essential equipment (e.g., computers, vehicles, motorcycles, sampling kits, protective tools, visual aid kits, tablets for data reporting, megaphones for awareness campaigns).

Funding (FID): Presence of a dedicated budget line for the OH platform to support routine and emergency activities.

Coordination (CID): Existence of formal intersectoral mechanisms for consultation, planning, and monitoring.

### Scoring and performance classification

Responses were coded using a standardized scoring system: 2 for “yes,” 1 for “partially,” and 0 for “no.” The scores for each indicator were aggregated and expressed as a percentage. Based on these scores, performance was classified into three categories: minimum (<60%), average (60%−80%), and best (above 80%). This performance assessment methodology, developed by Africa CDC, was adapted to evaluate monitoring indicators within the Guinean context.

### Data analysis

Descriptive statistics were first used to calculate the proportions of participants' socio-demographic characteristics. An integrated analytical approach was then applied, combining radar charts, correlation matrices, and dendrograms to provide a comprehensive understanding of the data. Radar charts enabled the visualization of regional performance across multiple indicators simultaneously, facilitating comparative analysis. A spatial map of Guinea was generated to display the performance scores for each administrative region, offering contextual insight and allowing for the identification of regional disparities and geographic priorities for targeted interventions. All statistical analyses and visualizations were conducted using R software, ensuring both the robustness of the calculations and high-quality graphical outputs.

### Ethics and confidentiality

The study received ethical approval from the National Committee of Ethics for Health Research (CNRS) in Guinea under reference number 025/CNERS//23. All data were handled confidentially and in accordance with applicable ethical standards.

## Results

### Participant profile

A total of 160 One Health platform actors were surveyed. The majority of respondents were male (77.2%), and 74.3% were between 26 and 45 years of age, reflecting an active working population within public services. Participants were primarily drawn from the three core sectors: human health (48.8%, *n* = 78), animal health (19.8%, *n* = 32), and environmental health (13.6%, *n* = 22). Additional stakeholders (18%) represented various public administration sectors (Table 1).

**Table 1:** Sociodemographic characteristics of participants.

**Category**	***n* (%)**
**Age group**	
19–25	6 (3.70)
26–35	53 (32.72)
36–45	62 (38.27)
46–55	25 (15.43)
56–65	9 (5.56)
65+	7 (4.32)
**Sectors**	
Elevage	32 (19.75)
Environment	22 (13.58)
Other	26 (16.05)
Partners	3 (1.85)
Health	79 (48.77)
**Gender**	
Female	37 (22.84)
Male	125 (77.16)

### Performance by indicator

The performance scores across indicators revealed substantial disparities (Table 2). The “Legislation” indicator showed the highest performance (77%), indicating the existence of formal regulatory and policy frameworks in most regions. Conversely, “Material Resources” recorded the lowest score (9%), highlighting widespread shortages in essential equipment such as vehicles, motorcycles, sampling tools, and personal protective equipment.

**Table 2:** Global performance indicators for surveillance in Guinea.

**Indicators**	**Expected score**	**Observed score**	**Performance (%)**
Legislation	644	495	77
Registration and detection of epidemics	1,288	763	59
Epidemic preparedness and response	2,898	1,476	51
Staff training	1,288	528	41
Material resources	2,576	243	9
Financing	322	172	53
Coordination	322	172	53
Overall score	9,338	3,849	41

**Figure 1:**
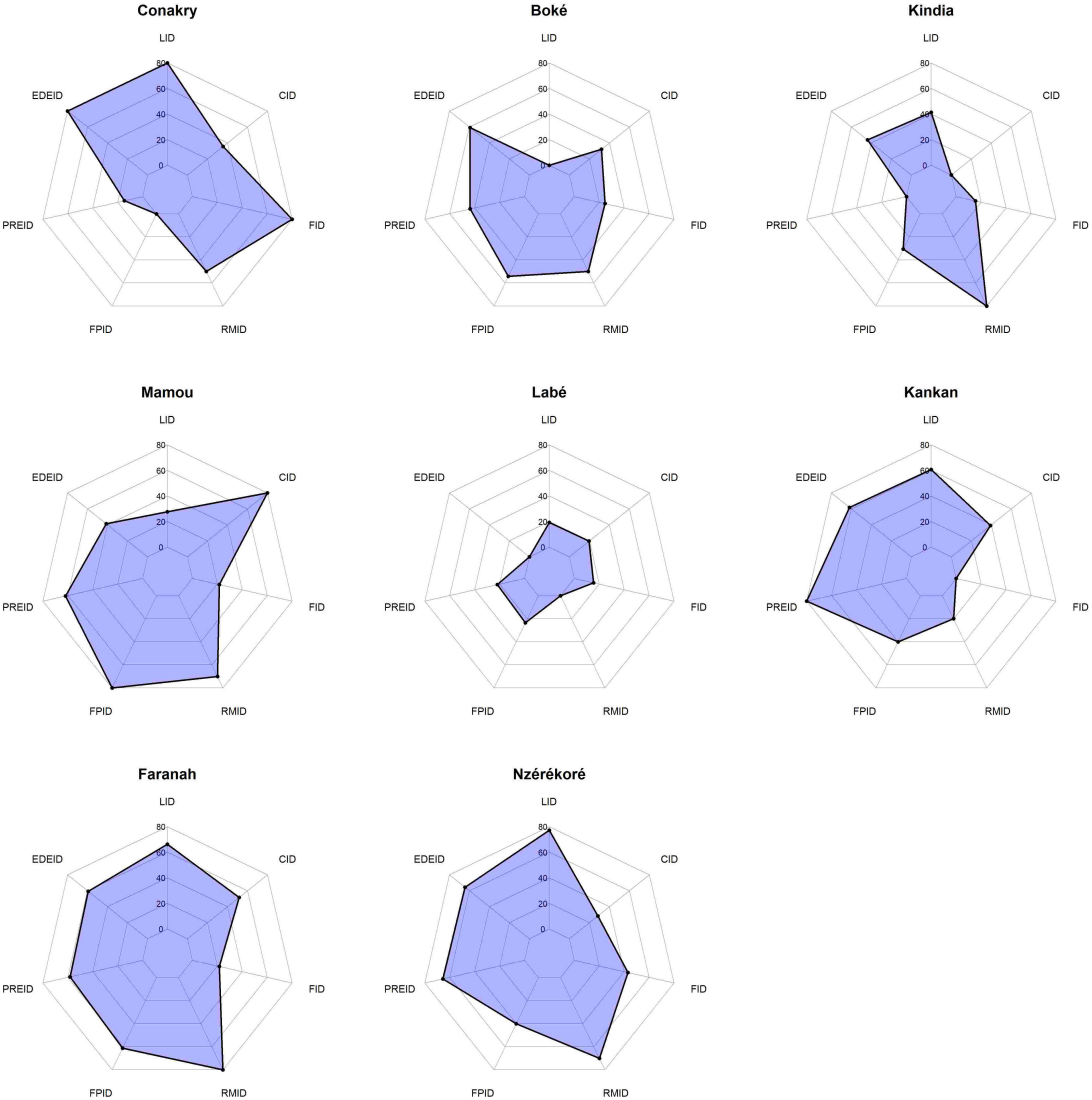
Distribution of performance indicators by region of Guinea. LID, legislation; EDEID, Registration and detection of epidemics; PREDID, epidemic preparedness and response; FPID, Staff training; RMID, material resources; FID, financing; CID, coordination.

The remaining indicators fell between these extremes: Coordination: 38%. Preparedness: 35%. Financing: 33%. Event Detection and Documentation: 32%. Training: 27%. These findings underscore a disconnect between the existence of regulatory texts and their effective operationalization (Figure 1).

### Regional comparison

The regional analysis showed marked heterogeneity in performance, with no region reaching the 60% threshold. Legislation was the only indicator to achieve relatively high scores (77%), particularly in Conakry, Kankan, Faranah, and N'zérékoré. In contrast, the mobilization of material resources was the most critical area, with an average score of 9%, particularly low in Conakry, Boké, Labé, and Kankan.

**Figure 2:**
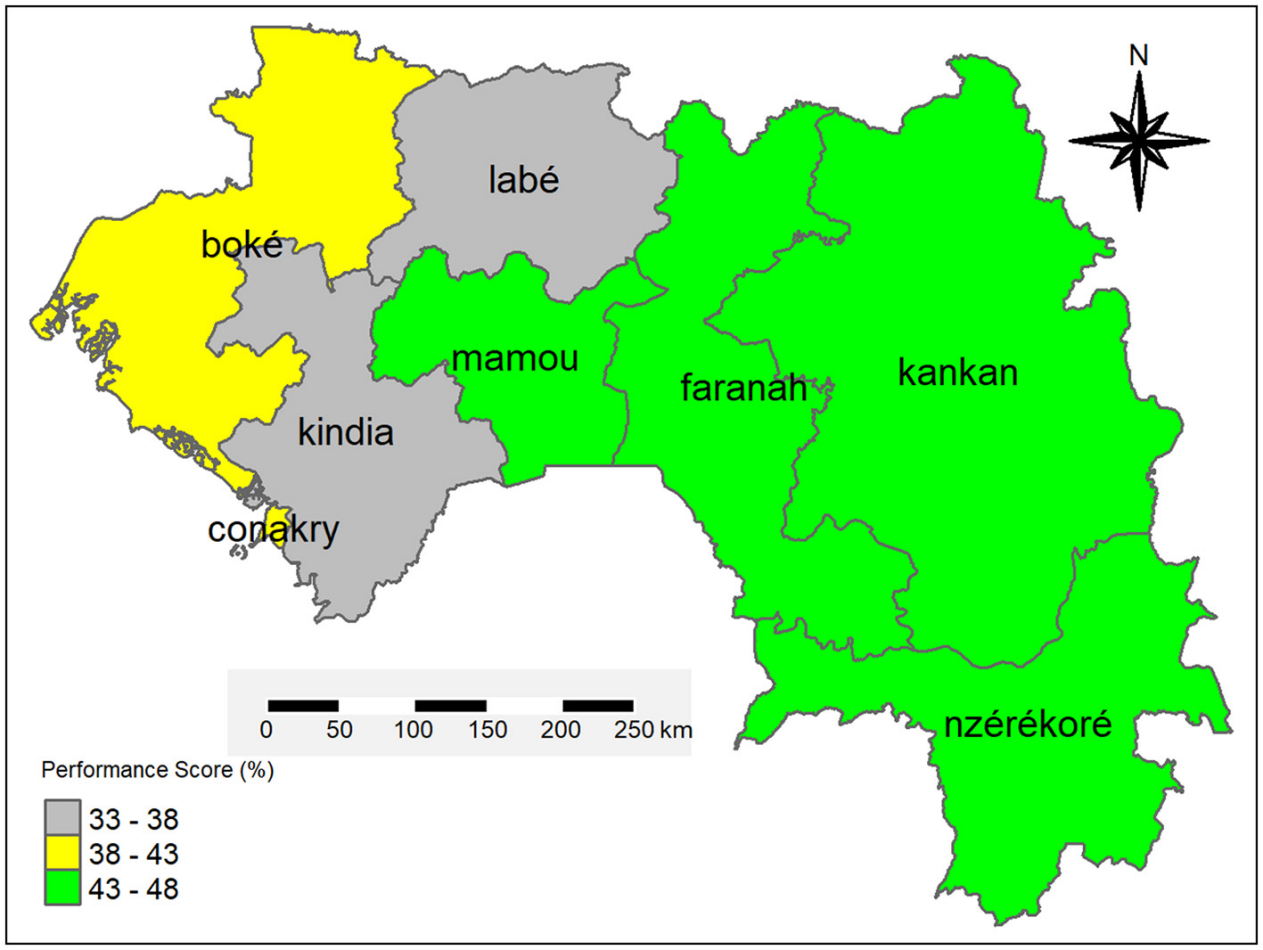
Overall performance of OH platforms in zoonoses surveillance by region of Guinea.

Overall scores ranged from 33% in Labé and Kindia to 46% in Faranah and Mamou, confirming systemic gaps and pronounced inter-regional disparities (Figure 2). All regional OH platforms in Guinea exhibited minimal performance (< 60%), underscoring the need to strengthen the operationalization of these platforms beyond mere institutional existence.

The disparities observed between regional One Health (OH) platforms in Guinea are largely attributable to differing geographical, socio-economic, and political contexts. Mamou, as the strategic crossroads connecting all other regions, benefits from enhanced monitoring of activities. Kankan, considered the country's second capital, receives strong political support and remains easily accessible. Faranah, due to its porous borders and the endemicity of Lassa fever, attracts considerable attention from partners, as does N'zérékoré, a region historically associated with major epidemic outbreaks. The latter benefits from close monitoring by both national authorities and technical and financial partners. In contrast, Kindia demonstrates relatively limited institutional engagement in OH platform coordination, while in Boké, actors' attention tends to be focused on mining-related priorities. These contextual factors help explain the performance disparities highlighted in this study.

## Discussions

The results revealed that all regional One Health platforms scored below 60%, placing them in the “minimum performance” category. The low performance observed across eight administrative regions of Guinea cannot be attributed solely to a lack of material and financial resources; rather, it reflects deeper systemic and institutional challenges. Chief among these are critical shortages in material and financial resources, which pose significant barriers to effective implementation. These findings align with those reported by Abdelmagid et al. (41), which demonstrated that limited resources in developing countries hinder the effective implementation of health programs. We noted the absence of continuous training and capacity building for involved actors, particularly in the area of epidemiological surveillance. Most of these individuals are civil servants, and upon retirement, they are often not replaced. These low scores can also be attributed to inconsistent strategy implementation, as highlighted by Seligsohn (42, 43) these low scores can also be attributed to inconsistent strategy implementation, as suggested in the study by Seligsohn on challenges encountered in field intervention programs. When replacements are made, the new personnel frequently do not receive the necessary training. Moreover, political changes at the national level have at times led to the replacement of trained staff with less qualified individuals. Finally, a lack of professional commitment among certain actors may also hinder the effective implementation of activities. These combined factors highlight the urgent need for institutional stabilization, the establishment of continuous training mechanisms, and the depoliticization of technical roles.

We recommend developing a national strategy for continuous training and institutional capacity building targeting One Health actors at all levels.

Although the overall performance of regional platforms remained generally low, some regions stood out with particularly high scores on specific indicators. Notably, areas such as legislation, coordination, and communication were particularly well implemented in certain regions, reaching up to 80%. These excellent performances suggest the presence of good practices or favorable conditions such as strong partner support, local leadership, or better institutional organization which should be identified, capitalized on, and replicated in other regional contexts.

For instance, N'zérékoré and Conakry achieved optimal performance in the legislation indicator, reflected by the availability of legal frameworks and a national monitoring manual for priority zoonoses. This success is likely due to targeted training efforts supported by local partners and NGOs, especially in N'zérékoré, where particular emphasis is placed on zoonosis surveillance. Such focused capacity building has significantly improved this indicator. These findings align with Rahman's research (44, 45), which highlights that targeted and ongoing training is a crucial factor in enhancing outcomes in public health programs. The use of GIS and digital tools presents a major opportunity to strengthen outbreak detection and better analyze regional disparities. However, their implementation depends on the availability of sufficient funding. It is therefore essential to mobilize sustainable technical and financial support to effectively integrate these technologies into One Health strategies.

Although most regions have low scores overall, notable differences exist when comparing specific performances. For example, in our study, the regions of N'zérékoré, Kankan, Faranah, and Boké each scored 60% on the indicator “Registration and detection of diseases,” which is classified as medium performance. These results suggest that these regions have implemented some of the necessary strategies for effective monitoring, but significant gaps remain to reach optimal performance. It was observed that most actors interviewed reported the use of standard case definitions, the capacity to transport samples, and the availability of guidelines for sample collection, handling, and transportation. This average performance may also be explained by factors such as incomplete training of health workers—particularly in the livestock and environmental sectors—and limited communication mechanisms between sectors involved in detecting zoonotic epidemics, as noted by Manageiro et al. (46). Studies by Morse et al. (47) have shown that, in similar contexts, insufficient coordination between the human, animal, and environmental sectors often limits the effectiveness of early epidemic detection mechanisms (48). In addition, research such as those by Zhang et al. (22) shows that technologies such as geographic information systems (GIS) and digital tools could significantly improve the capacity to record and detect outbreaks in regions (49, 50).

Analyzing the performance across the eight regions, N'zérékoré, Kankan, Faranah, and Boké achieved a performance score of 60%, while the other four regions (Labé, Mamou, Kindia, and Conakry) recorded scores below 60%, placing them in the minimum performance category for outbreak detection and recording. These results highlight significant challenges in epidemic detection within these regions. The low performance observed could be attributed to systemic deficiencies. This is consistent with previous studies, such as those by Nana et al. (49), which emphasize that structural limitations often hinder effective disease surveillance (49, 50), have shown that low-income regions often suffer from a lack of intersectoral coordination, an absence of early warning mechanism, and a limited capacity for data collection and analysis, mainly in the livestock and environmental sectors (51). Performance disparities among regional One Health platforms in Guinea can also be attributed to structural determinants that are well recognized by field actors. Mamou, as a strategic national transit hub, benefits from enhanced oversight due to its central geographic position. Kankan, the country's second administrative center, enjoys consistent political support and good logistical accessibility. Faranah, owing to its porous borders and the endemic circulation of Lassa fever, remains under close surveillance by technical partners. N'zérékoré, historically associated with major epidemic outbreaks, receives sustained technical support from both national authorities and external partners, within a framework of prevention and pilot testing of integrated approaches. In contrast, Kindia suffers from limited institutional engagement in coordinating its OH platform, while Boké remains largely focused on economic priorities linked to mining activities, particularly bauxite. These often-overlooked contextual realities are key to understanding the observed performance gaps and should inform targeted strengthening efforts. These factors were also observed firsthand during our interviews with stakeholders on the ground. Our study identified challenges related to continuing education and insufficient human resources, particularly in the livestock and environmental sectors. Additionally, technological limitations and budgetary constraints were highlighted. Nana et al. (49), in her study conducted in Burkina Faso, similarly reported the absence of dedicated budget lines to support One Health platform activities (49). For instance, during our interviews, actors confirmed the lack of access to office equipment, insufficient financial incentives following field investigations, and inadequate ongoing training in surveillance and epidemiology, especially within the livestock and environmental sectors. Compounding these issues are widespread retirements without replacement and political changes that have not been capitalized on. New personnel often lack training in disease surveillance and an understanding of the One Health approach. However, a comparison with higher-performing countries, as illustrated by Singh et al. (52), showed that implementing continuous training programs, improving information systems, and strengthening cross-sector collaboration could transform these weaknesses into opportunities (53).

These findings highlight an urgent need to strengthen the operational capacities of One Health (OH) platforms across all regions of Guinea. The fact that every region exhibited minimal performance underscores systemic challenges rather than isolated regional weaknesses, calling for a coordinated national strategic response. This study fosters a collective reflection on actionable pathways to improve OH platform performance. The lessons drawn from this evaluation can inform the development of targeted improvement strategies, particularly through increased support from international partners and the integration of digital solutions into the disease surveillance framework.

It should be noted that the sample size (*n* = 160) accurately reflects the actual composition of the existing regional platforms. Participants were purposively selected based on their effective involvement within the platforms, ensuring functional rather than statistical representativeness. This approach is widely recognized and commonly used in institutional assessments conducted in resource-limited settings, where the number of relevant actors is structurally constrained.

Second, the cross-sectional nature of this study restricts its ability to capture temporal dynamics or establish causal relationships. The performance scores presented herein reflect a snapshot in time (May, June 2023). Longitudinal or repeated assessments would offer better insight into the progression or regression of OH platforms over time.

Third, despite efforts to adapt the data collection tools, potential interpretation biases may persist, particularly due to the subjective self-reporting by participants. Mitigating measures included the individual administration of questionnaires and the training of data collectors to ensure consistency and reduce bias.

Finally, although the study covered all eight administrative regions of Guinea, sub-regional disparities at the level of prefectures or sub-prefectures may not have been fully captured. Nonetheless, the findings offer a reliable overview of the structural strengths and weaknesses of regional OH platforms and provide a solid foundation for guiding national-level decision-making and resource allocation.

As part of future research, the integration of the Global One Health Index (GOHI) could enhance the comparative analysis of OH platform performance, allowing for comparisons not only at the regional and national levels but also against international standards. The use of this framework would help better position Guinea's efforts in combating zoonoses on the global stage and strengthen accountability mechanisms.

## Conclusion

This study assessed the performance of regional One Health (OH) platforms in Guinea in the context of zoonotic disease surveillance, using a set of key indicators. The findings reveal a generally low and homogeneous level of performance across regions, although some regions scored higher on specific indicators such as legislation and coordination. These disparities reflect recurrent structural weaknesses, including limited intersectoral coordination, insufficient technical and human capacity, and inadequate access to material and financial resources.

Despite these shortcomings, the study highlights the significant potential of OH platforms if targeted capacity-building efforts are undertaken. Strengthening collaboration mechanisms across sectors and enhancing the competencies of key actors are critical levers to improve platform effectiveness. This research contributes to the growing body of literature on the operationalization of the One Health approach in resource-limited settings.

## Data Availability

The raw data supporting the conclusions of this article will be made available by the authors, without undue reservation.
